# Transcriptome and N6-Methyladenosine RNA Methylome Analyses in Aortic Dissection and Normal Human Aorta

**DOI:** 10.3389/fcvm.2021.627380

**Published:** 2021-05-28

**Authors:** Xianwu Zhou, Zerui Chen, Jianrong Zhou, Yaorong Liu, Ruixin Fan, Tucheng Sun

**Affiliations:** ^1^Department of Cardiovascular Surgery, Guangdong Cardiovascular Institute, Guangdong Provincial Key Laboratory of South China Structural Heart Disease, Guangdong Provincial People's Hospital, Guangdong Academy of Medical Sciences, Guangzhou, China; ^2^Department of Cardiovascular Surgery, Zhongnan Hospital of Wuhan University, Wuhan, China

**Keywords:** aortic dissection, transcriptome analysis, N6-methyladenosine RNA methylome analysis, extracellular matrix, inflammatory responses three

## Abstract

**Objective:** To investigate the N6-methyladenosine (m6A) modification and the expressions of the m6A regulatory genes in the acute aortic dissection (AD).

**Methods:** MeRIP-seq and RNA-seq experiments of aortic media tissue samples obtained from AD (*n* = 4) and Controls (*n* = 4) were conducted. m6A methylation quantification was used to measure the total mRNA m6A level. The five m6A regulators mRNA expressions were analyzed by quantitative polymerase chain reaction (qPCR). Western blot analyses and immunofluorescence staining were used to detect the difference of METTL14 protein expression in the aortas of AD and Normal.

**Results:** Among AD patients, we detected significantly elevated levels of m6A in total RNA. Compared with the normal group, the up methylated coding genes of AD were primarily enriched in the processes associated with extracellular fibril organization, while the genes with down methylation were enriched in the processes associated with cell death regulation. Furthermore, many differentially methylated m6A sites (DMMSs) coding proteins were mainly annotated during the extracellular matrix and inflammatory responses.

**Conclusions:** These findings indicate that differential m6A methylation and m6A regulatory genes, including MTEEL14 and FTO, may act on functional genes through RNA modification, thereby regulating the pathogenesis of aortic dissection.

## Introduction

Acute Stanford type A aortic dissection (AD) is an extremely dangerous cardiovascular disease that is associated with a high mortality rate. With the improved living standards and enhanced detection methods, the incidence rate remains increasing, but the pathogenesis is still unclear. AD is most commonly seen in individuals aged 65–75 years old, with 35 cases per 100,000 people each year (1). So, etiological exploration is much necessary to reduce the incidence of AD and improve the clinical outcome.

Although DNA methylation is potentially an important mechanism in heart failure (2) and atherosclerotic lesions (3), the role of RNA modification in aortic disease has not been studied in a detailed manner. Increasing evidence indicated that abnormal gene expression that is regulated by transcription factors and epigenetic processes (such as non-coding RNA, DNA, and histone modifications) is a key incident in aortic dissections and aortic aneurysms (4, 5), which might in turn act as new therapeutic interventions.

N6-methyladenosine (m6A) is an epigenetic RNA modification that occurs in most eukaryotes. Recent studies have identified multiple proteins that are associated with m6A in the regulation of blood pressure (6), cardiac gene expression and cell growth (7), cell survival, and intracellular signaling (8). Previous studies have demonstrated the functional importance of cardiac Fat mass and obesity-associated protein (FTO)-dependent m6A methylome during myocardial infarction (9) and cardiac growth control by Methyltransferase Like 3 (METTL3) (10). Compared with normal aortic tissues, the relative methylation level of m6A genes and the number of genes that is associated with m6A methylation in abdominal aneurysmal tissues have been significantly increased. The protein expression level of m6A methylases [such as Methyltransferase Like 14 (METTL14), FTO, YT521-B homology F1 (YTHDF1) and YT521-B homology F3 (YTHDF3)] in abdominal aortic aneurysmal tissue samples has been significantly increased. Among these, FTO and YTHDF3 were localized in vascular smooth muscle cells (VSMCs), and the number of VSMCs has a positive correlation with FTO expression (11).

To our knowledge, m6A modified mRNAs have not been addressed experimentally in AD. To investigate the differences in the m6A modification patterns between AD and normal healthy aorta, and to explore the relationship between m6A modification and extracellular matrix degradation, an m6A-specific RNA immunoprecipitation sequencing (MeRIP-seq) of normal aorta and AD with high-throughput sequencing was performed. In general, when compared with the normal aorta, 195 differentially methylated peaks within the mRNAs were detected. More interestingly, our study characterized the coding genes harboring the differentially methylated peaks that were involved in many important biological pathways in association with the extracellular matrix, thereby providing a target to improve extracellular matrix homeostasis in aortic diseases.

## Methods

### Ethics Statement and Samples

This study was approved by the Ethics Committee of Guangdong Peoples' hospital and all patients provided written informed consent. During surgery, the samples of ascending aortas were obtained safely. There were no known potential confounders during sampling of clinical specimens and tissues were immediately snap-frozen and kept in liquid nitrogen for later use. Eight samples were collected, which included four patients with acute ascending aortic dissection (AD), and four from excess donor aortic tissue during heart transplantation (Normal). The cause of the donor's death was cerebrovascular or motor vehicle accident. All donors had no history of cardiovascular diseases or active infection at the time of transplantation. Acute aortic dissection is defined as dissection detected within 2 weeks of the onset of symptoms, and aortic repair was performed within 48 h of symptom onset in all four patients. All subjects in this study did not include hereditary aortic diseases, connective tissue disorders, cancers, drug history, infections, or any other immune-related diseases that may affect the study.

### RNA Preparation, Library Construction, and Sequencing

Total RNA from the tissue was extracted using TRIzol reagent (Invitrogen Corporation, CA, USA) following the manufacturer's instructions. The RNA was then fragmented chemically into about 100–200 nucleotides in length using fragmentation buffer (Illumina, Inc.). The RNA was precipitated using the aqueous phase by mixing with isopropyl alcohol. The samples were incubated at 15°C to 30°C for 10 min and centrifuged at 12,000 x g for 10 min at 4°C.

Next, the cytoplasmic ribosomal RNA and mitochondrial rRNA were removed from the total RNA by probe hybridization and enzymatic hydrolysis using the Ribo-Zero rRNA removal kit (Illumina, CA, USA). The ribosomal RNA-depleted RNAs were then used to construct RNA libraries with TruSeq Stranded Total RNA Library Prep kit (Illumina, CA, USA). The strand-specific library construction uses dUTP instead of dTTP to label the second-strand cDNA during synthesis. Uracil-DNA glycosylase (UDG) was used to hydrolyze the strand before the polymerase chain reaction (PCR) enrichment to ensure that the final sequencing data was from the first cDNA strand. Besides, actinomycin D was added to the first cDNA strand synthesis to inhibit the binding of reverse transcriptase to the DNA template and to avoid the synthesis of a pseudo-negative strand, enhancing the strand specificity of the sequencing data. The protocol for strand-specific library construction included:

RNA fragmentation: (1) First-strand cDNA synthesis; (2) Second strand cDNA synthesis; (3) End Prep/dA-tailing of cDNA Library; (4) Adaptor ligation, and (5) PCR enrichment of adaptor-ligated DNA.

The library quality was assessed using a Bioptic Qsep 100 analyzer. The electropherogram showed a narrow distribution with a peak size of approximately 350–400 bp. The libraries were with paired-end sequences with reading lengths of 150. To remove the 3' adaptor-trimming and low-quality reads with cut-adapt software (v1.9.3), the high-quality trimmed reads were aligned to the reference genome (hg38) with HISAT2 software (v2.0.4). Read counts was calculated using HTSeq (v0.10.0). The cuffdiff software (v2.2.1) was then used to obtain the expression profiles of mRNAs in terms of fragments per kilobase of transcript per million fragments mapped (FPKM). The DEGs were assessed using the DESeq2 R package (v1.10.1) between the AD group and the normal individuals. Screening was performed under the threshold of FDR < 0.05 and |log2FC| > 1.

### RNA MeRIP-Seq Library Construction & Sequencing

In brief, the RNA fragments were incubated with anti-N6-methyladenosine (m6A) antibodies (Sigma-Aldrich, Burlington, USA) immunoprecipitation (IP) buffer for 2 h at 4°C. The samples were then washed with low-salt precipitation buffer and high-salt buffer thrice continuously. RNA was extracted using a phenol-chloroform lysate to obtain a purified product. The purified RNA was collected for the generation of an RNA-seq library with NEBNext R Ultra™ RNA Library Prep Kit (New England Biolabs, MA, USA). The quality of the library was done using a Bioptic Qsep100 analyzer and sequencing was done using NovaSeq's high-throughput sequencing platform. The quality of the paired-end reads was analyzed using fastqc (v0.11.8), and the clean reads of all libraries were aligned in accordance with the reference genome (hg38) using Hisat2 software (v2.0.4) (12). Differential methylation sites were identified using the exomePeak R package (v2.13.2) with default parameters.

### Quantitative Reverse Transcription PCR (qRT-PCR) and Total mRNA m6A Level Determination

qRT-PCR was performed using Universal SYBR qPCR Master Mix (Q711-02/03, Vazyme Bio) after the synthesis of cDNA using the SYBRR Premix Ex Taq (TaKaRa, Japan). qRT-PCR analysis for all samples was independently repeated at least twice. Normalization was performed with GAPDH. The RNA specific primers were listed in Supplementary Table 1. The EpiQuik m6A RNA Methylation Quantification Kit (Colorimetric) (P-9005, Epigentek, USA) was used to measure the m6A content in total RNAs.

### Western Blot Analyses

Total protein was extracted using a commercial kit (Protein Extraction Kit, Millipore) and 30 μg total protein from each aorta sample was loaded onto subjected to 12% sodium dodecyl sulfate-poly-acrylamide gel electrophoresis (SDS-PAGE) using a 10% running gel, dissociated with 10% SDS-PAGE, and probed with primary antibodies for METTL14 (Abcam, ab223090; 1:500 dilution) and β-actin (Abcam ab8227; 1:1,000 dilution) at 4°C overnight. Next, the membranes were washed and incubated with an HRP-conjugated anti-rabbit secondary antibody (Abcam ab5694; 1:5,000 dilution) for 2 h. Bands were detected using enhanced chemiluminescence (ECL Advance; #WBKLS0500, Millipore Corporation). The signals were recorded using a ChemiDoc imaging system (Bio-Rad Labora-tories) and analyzed with ImageJ analysis software (NIH, Bethesda, MD, USA).

### Immunofluorescent Staining

AD and Normal aortas were subjected to immunofluorescent staining. Frozen slides of 5 mm sections were fixed with acetone, blocked with 1% bovine serum in PBS for 1 h, and incubated at 4°Covernight with primary anti-METTL14 (Abcam, ab223090; 1:1,000 dilution). Then, the slides were incubated with fluorescence-labeled secondary antibodies at 37°C for 30 min in the dark. To every slide, 50 μL of DAPI (Abcam, ab104139; 1:500 dilution) was added. After 10-min incubation, the slides were washed three times with PBS, and images were obtained with a fluorescence inverted microscope (Olympus BX51, Tokyo, Japan).

### Data Analysis

The comparison between the two groups was performed using the GraphPad software with an unpaired Student's *t*-test. ^*^P < 0.05 was considered statistically significant.

### Independent Data Access

The RNA-seq and MeRIP-seq data has been deposited in GEO under accession code GEO: GSE147028.

## Results

### Characterization of Study Subjects

The demographic and clinical characteristics of AD patients and controls included in this study are shown in Table 1. There were no statistically significant differences in clinical characteristics between the AD and Normal groups, except for hypertension.

**Table 1:** Clinical characteristics of the AD and normal groups.

**Characteristics**	**AD group (*n* = 4)**	**Normal group (*n* = 4)**
Age (year)	54.25 ± 3.88	47.00 ± 5.18
Male (%)	2 (50)	2 (50)
Smoker (%)	1 (25)	1 (25)
Alcoholic (%)	0 (0)	0 (0)
Hypertension (%)	4 (100)^*^	1 (25)
Atherosclerosis (%)	0 (0)	0 (0)
Cardiac disease (%)	0 (0)	0 (0)
Cocaine abuse (%)	0 (0)	0 (0)
Diabetes (%)	0 (0)	0 (0)
Bicuspid aortic (%)	0 (0)	0 (0)
Medication use (%)	0 (0)	0 (0)

*AD, acute aortic dissection. Data are presented as a mean ± SD or n (%)*.

* *p < 0.05. The demographic and clinical characteristics of AD patients and controls included in this study are shown in the table. There were no statistically significant differences in clinical characteristics between the AD and Normal groups, except for hypertension*.

### The Expression of m6A Level and m6A Methylation Regulatory Genes in AD

**Figure 1:**
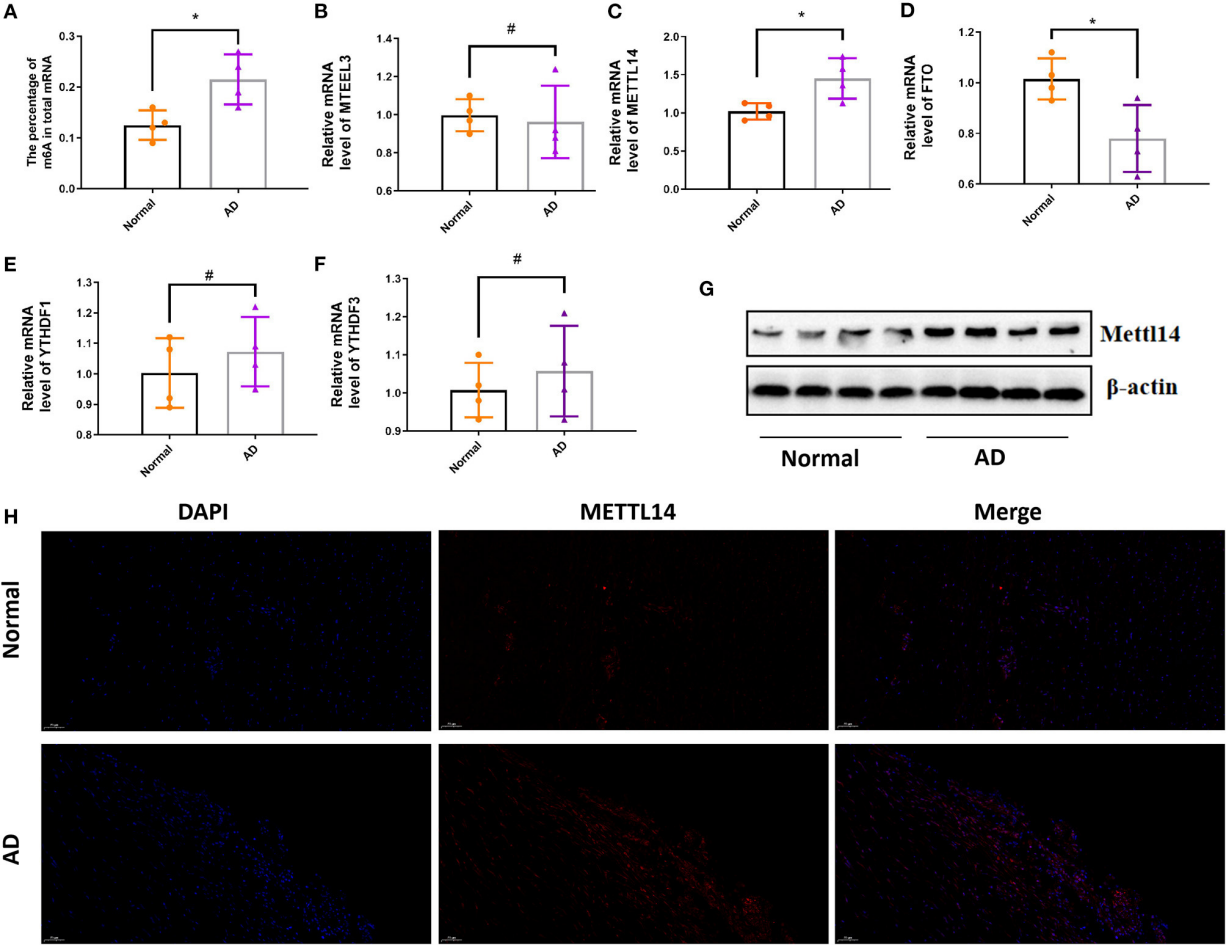
The expression of m6A level and m6A methylation regulatory genes in AD. The mRNA m6A level and m6A methylation regulatory genes at the mRNA level in AD compared with the normal aortas. **(A)** The mRNA m6A level in AD and normal aortas; **(B–F)** The mRNA expression of METTL3, METTL14, FTO, YTHDF1 and YTHDF3 in AD and normal aortas. GAPDH was used as an internal control. **(G)** Western blot analysis of the METTL14 in AD and normal aortas. All of the results represent the mean ± standard deviation of three independent experiments (*n* = 4). **(H)** Representative immunofluorescent staining of METTL14, and 4',6-diamidino-2-phenylindole (DAPI) in AD and normal aortas (scale bars, 50 μm). *P < 0.05, ^#^*P* > 0.05.

By the EpiQuik m6A RNA Methylation Quantification Kit, we found the percentage of m6A in total mRNA in AD tissue samples was higher than that in the normal aortic tissue samples (Figure 1A). We then determined the mRNA expression levels of several regulatory genes participating in m6A mRNA modification. The expression level of METTL14 was significantly up-regulated, while FTO expression was significantly down-regulated in AD tissue samples compared with normals (Figures 1C,D). There was no significant difference in the expression levels of the other genes, including METTL3, YTHDF1, and YTHDF3 (Figures 1B,E,F). At the same time, we detected the protein expression level of METTL14 and found that compared with normals people, AD tissue samples were significantly up-regulated (Figures 1G,H).

### DEGs Clustering and Functional Enrichment

**Figure 2:**
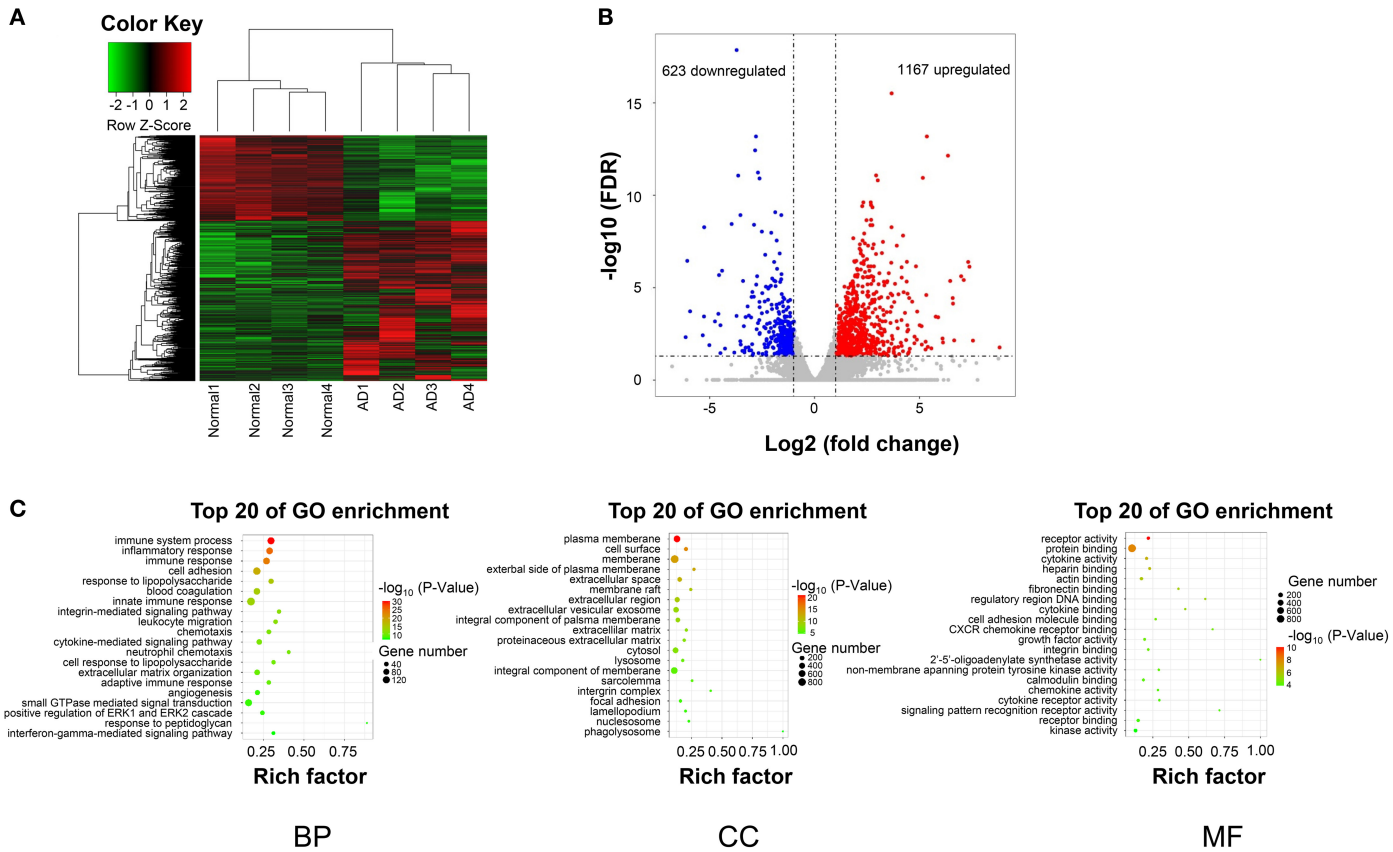
DEGs hierarchical clustering analysis and functional annotation. **(A)** Heatmap of DEGs. The vertical axis represents DEGs. The horizontal axis represents the sample. DEGs are effectively divided into AD and normal groups. Red indicates upregulated genes and green represents downregulated genes. **(B)** The volcano plot of DEGs. The vertical axis represents -log 10 False Discovery Rate (FDR) and the horizontal axis represents log 2 fold change (log2FC). The DEGs were selected according to FDR < 0.05 and |log2FC| > 1, and the number of upregulated genes is 1167 and the number of down genes is 623. The blue and red dots represent DEGs and black dots represent nondifferential expressed genes. **(C)** The top 15 enriched GO terms (biological process, cellular component, and molecular function) of the DEGs, respectively.

After data preprocessing and quality assessment, a total of 1,790 differentially expresssd genes (DEGs) (1,167 upregulated and 623 downregulated) were filtered out (Figures 2A,B).

The biological significance was explored by GO categories enrichment analysis, such as the biological process (BP), cellular component (CC), and molecular function (MF), to reveal the AD-related functional annotated terms. The GO enrichment analysis of 1,790 DEGs showed that 1,167 upregulated genes, and 623 downregulated genes (Figure 2C). The most enriched terms of biological processes were mainly included in the immune system process, inflammatory response, and cell adhesion in the AD group (Figure 2C).

GO analysis demonstrated the significantly enriched DEGs in BP, including immune system process, inflammatory response, cell adhesion (Figure 2C); CC, including the plasma membrane, cell surface, membrane (Figure 2C); and MF, including receptor activity and protein binding (Figure 2C).

### Distribution of Differentially Methylated m6A Sites

**Figure 3:**
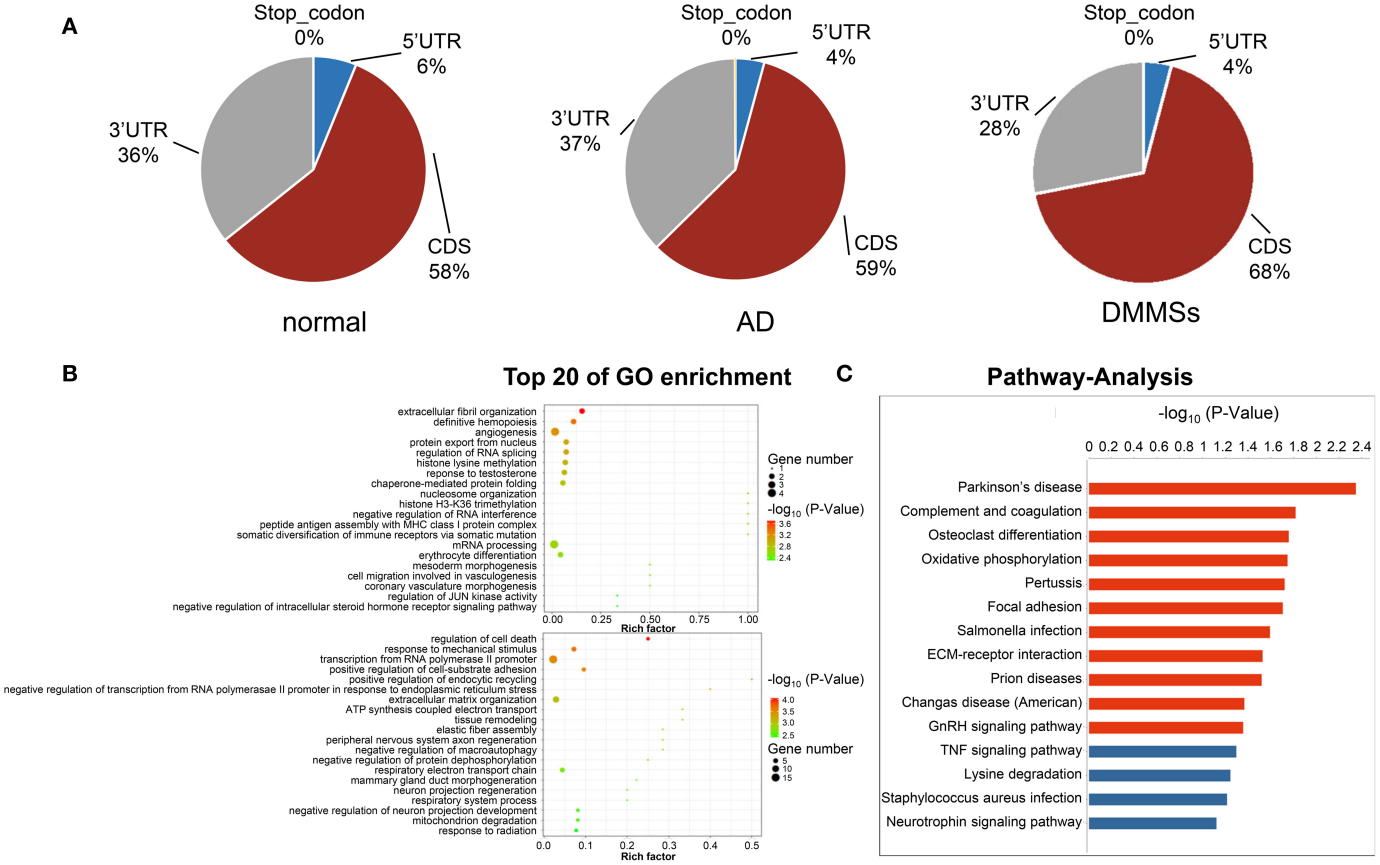
Differentially methylated N6-methyladenosine sites. **(A)** Pie charts showing the percentage of m6A peaks in five non-overlapping segments of transcripts in normal aorta, aortic dissection (AD) and differentially methylated m6A sites (DMMSs). m6A peaks were mostly enriched in the coding sequence segment. **(B)** The top twenty gene ontology terms of biological processes showed significant enrichment of up-methylated and down-methylated genes. **(C)** Bar plot showing the top ten enrichment scores of the significant enrichment pathway for the up- or down-methylated genes.

In total, 195 differentially methylated m6A sites (DMMSs) within 179 nuclear coding genes were identified, and 80.5% (157/195) of these were significantly down methylated sites (AD vs. normal; Table 2). Tables 3, 4 showed that the top ten up and down methylated m6A sites within the mRNAs have the highest fold change values, which were >100-fold. All DMMSs within the mRNAs were mapped to chromosomes and the top 4 chromosomes harboring the most DMMSs were 11 (25), 19 (24), 1 (21), and 2 (13). We further analyzed the total m6A distribution patterns of mRNAs according to the m6A-seq results. Similar patterns of the total m6A distribution in Normal and AD groups were observed (Figure 3A).

**Table 2:** General numbers of differentially methylated peaks and associated genes.

**Item**	**Upmethylated peak**	**Upmethylated gene**	**Downmethylated peak**	**Downmethylated gene**
mRNA	38	35	157	144

*In total, 195 differentially methylated m6A sites (DMMSs) within 179 nuclear coding genes were identified, and 80.5% (157/195) of these were significantly down methylated sites*.

**Table 3:** Top 10 up methylated m6A peaks.

**Chromosome**	**txStart**	**txEnd**	**Gene name**	**Log2-fold change**
8	80487325	80487596	ZBTB10	11.1
11	73309428	73309789	ARHGEF17	10.7
19	49108276	49108545	SNRNP70	10.6
11	78218411	78218862	GAB2	10.1
3	47120601	47120932	SETD2	8.91
7	77196647	77196976	FGL2	8.77
15	75676655	75676866	CSPG4	8.24
14	75469405	75470035	JDP2	8.12
19	55616700	55617000	ZNF865	8.08
11	6627542	6627663	DCHS1	7.67

*txStart/txEnd: Start/end position of the differentially methylated RNA peaks. The top 10 up methylated m6A sites within the mRNAs have the highest fold change values, which were >100-fold*.

**Table 4:** Top 10 down methylated m6A peaks.

**Chromosome**	**txStart**	**txEnd**	**Gene name**	**Log2-fold change**
X	103377507	103377687	BEX3	−17.4
1	160214331	160214511	PEA15	−13.2
15	48878153	48878304	EID1	−13
20	3785255	3785435	CENPB	−12.9
6	26124442	26124651	HIST1H2AC	−12.3
1	230281435	230281616	GALNT2	−11.7
7	5622805	5623046	RNF216	−10.8
19	38291896	38292076	SPINT2	−10.8
2	206056634	206056815	INO80D	−10.7
1	204423766	204423947	PIK3C2B	−10.7

*txStart/txEnd: Start/end position of the differentially methylated RNA peaks. The top 10 down methylated m6A sites within the mRNAs have the highest fold change values, which were >100-fold*.

### Differentially Methylated RNAs Are Involved in Important Biological Functions and Pathways

To uncover the functions of m6A in AD, the protein-coding genes containing DMMSs were selected for GO enrichment analysis (Figure 3B) and KEGG pathway analysis (Figure 3C). For the BP category, the genes with up or down methylated m6A sites showed significant (*p* < 0.05) enrichment in regulating the transcription, while genes with down methylated m6A sites also showed high enrichment in the apoptotic process. For the CC category, genes with up methylated m6A sites were mainly enriched in the nucleus, while down methylation of m6A was enriched in the extracellular matrix. For the MF category, both up or down methylation of m6A sites showed notable enrichment in protein binding.

These results suggested that m6A might play an important role in the occurrence and development of AD, with the primary roles in the extracellular matrix and transcriptional regulation using DNA as a template. For example, tenascin C (TNC) showed association with the extracellular matrix organization and positive regulation of cell proliferation, wherein m6A was up methylated (AD vs. normal) (Table 5), while fibulin 5 (FBLN5) is associated with elastic fiber assembly, wherein m6A was down methylated (Table 6).

**Table 5:** DMM overlap with up-regulated DEGs.

**Gene name**	**Gene symbol**	**gene ontology terms of biological processes (selected)**	**mRNA regulation Log2-fold change**
apolipoprotein E	APOE	response to reactive oxygen species	2.963896
tenascin C	TNC	positive regulation of cell proliferation	2.817838
solute carrier family 12 member 8	SLC12A8	ion transport	2.447643
JunB proto-oncogene	JUNB	vasculogenesis	1.9883
adrenomedullin	ADM	vasculogenesis	1.909539
elastin microfibril interfacer 1	EMILIN1	aortic valve morphogenesis	1.54932
pleckstrin homology domain containing A4	PLEKHA4	phosphatidylinositol biosynthetic process	1.544703
ras homolog family member G	RHOG	actin filament organization	1.415065
matrix metallopeptidase 14	MMP14	angiogenesis	1.358464
complement C1r	C1R	regulation of complement activation	1.357514
ribosome binding protein 1	RRBP1	osteoblast differentiation	1.253301
protein phosphatase 1 regulatory subunit 18	PPP1R18	cytoplasm	1.061758

*DMM, differentially methylated N6-methyladenosine; DEGs, differential expressed genes. Differentially methylated N6-methyladenosine overlap with up-regulated differential expressed genes. Multiple genes are involved in the positive regulation of extracellular matrix organization and cell proliferation*.

**Table 6:** DMM overlap with down-regulated DEGs.

**Gene name**	**Gene symbol**	**gene ontology terms of biological processes (selected)**	**mRNA regulation Log2-fold change**
SPARC like 1	SPARCL1	cellular protein metabolic process	−1.92692
transmembrane protein 30B	TMEM30B	aminophospholipid transport	−1.57251
dual specificity phosphatase 1	DUSP1	inactivation of MAPK activity	−1.56709
zinc finger and BTB domain containing 10]	ZBTB10	nucleoplasm	−1.40601
fibrinogen like 2	FGL2	immunoglobulin production involved in immunoglobulin mediated immune response	−1.39772
protocadherin 7	PCDH7	cell adhesion	−1.35683
Kruppel like factor 9	KLF9	regulation of transcription by RNA polymerase II	−1.31683
fibulin 5	FBLN5	extracellular matrix organization	−1.24061
kinesin family member 5B	KIF5B	cytoplasm organization	−1.06699
purine rich element binding protein A	PURA	DNA replication initiation	−1.04003

*DMM, differentially methylated N6-methyladenosine; DEGs, differential expressed genes. Differentially methylated N6-methyladenosine overlap with down-regulated differential expressed genes. Multiple genes are associated with elastic fiber assembly and immune response*.

KEGG pathway analysis of DMMS-contained mRNA-associated genes was performed and the top twenty pathways with the highest enrichment score (-log10, *p*-value) are presented in Figure 2C. Some genes were involved in extracellular matrix–receptor interaction, such as Collagen Type VI Alpha 2 Chain (COL6A2), while others were involved in complement and coagulation cascades, such as Complement Component 1, R subcomponent (C1R).

## Discussion

AD is currently a serious public health problem worldwide and patients with AD are associated with extremely high mortality rates. Convincing evidence has suggested that VSMCs play a key role in the development and progression of AD. Investigation of the underlying mechanism of VSMCs in AD assists in the intervention of a new therapeutic strategy.

Previous studies have focused on the signaling pathways of VSMCs proliferation activation, apoptosis, and phenotypic transition (13, 14). Although significant progress has been made in understanding the transcriptional regulation of gene expression during AD formation, m6A methylation plays an important and diverse biological function during the process of apoptosis. M6A dynamically regulated the cell responses to environmental stress, including apoptosis and oxidative stress (14). The underlying mechanisms of how m6A RNA regulates AD formation are still unclear. Chen et al. (15). have reported that m6A mRNA modification also affected vascular calcification, but there is a lack of research on m6A in the cardiovascular field. In this research, we first demonstrated that in AD tissue samples there was an increasing percentage of mRNA modified by m6A relative to healthy aortic samples, and conjoint analysis of m6A-RIP-seq and RNA-seq data has identified m6A-modified mRNA transcripts that were also significantly differentially expressed. Based on the potential role of m6A modification in the pathophysiology of AD, correction in the changes in m6A levels might also be a promising strategy.

To detect regulatory factors that may be involved in m6A modification, we subsequently analyzed the mRNA expression of several important genes. The mRNA and protein expression level of METTL14 was up-regulated, while the mRNA level of FTO was down-regulated in AD tissue samples compared with Normals. In the previous study, the differences among the expressions of METTL14 and FTO were found in the different types of inflammatory cells that exist in the AD tissues (16) and m6A mRNA modification can regulate T cell homeostasis (17). Based on these findings, we surmise that m6A modification may have an impaction on the expressions of genes in different types of inflammatory cells, which cooperatively regulate AD progression.

m6A modification modulates all stages in the life cycle of RNA, such as RNA processing, nuclear export, and translation modulation (18). In the early years, the research of m6A mainly focused on the methylation modification at 5'Cap and 3'polyA of mRNA. Its functions include maintenance of mRNA stability, mRNA precursor splicing, polyadenylation, mRNA transport and translation initiation, etc. Recently, it has been found that the increased expression of m6A in coding sequence (CDS) can also promote translation of target gene mRNA (19). In this study, most of the DMMSs within the mRNAs were within a CDS. Is the m6A methylation change in the CDS region of the mRNA one of the reasons for the occurrence of aortic dissection? This requires more gain and loss of function studies.

Evidence (20, 21) have indicated that there is a close relationship between m6A modification and the extracellular matrix, revealing the importance of m6A in the extracellular matrix. As observed, it is interesting that the results of GO and KEGG analyses regarding the genes encoding DMMSs indicated that methylated genes are mainly concentrated in the processes and pathways related to cellular matrix and elastin functions, such as elastic fiber assembly, the extracellular matrix organization, and ECM-receptor interaction signaling pathways (Figure 3C). This in turn supported the importance of m6A in the cellular matrix changes. For example, in this study, fibulin 5 (FBLN5) has enriched the extracellular matrix organization, and the content of FBLN5 was reduced by about half when compared with the normal group. FBLN5 helps in maintaining the integrity of the vessel wall after injury and avoiding abnormal remodeling. In the oxidative stress response, reactive oxygen species inhibited the binding of FBLN5 to elastin by blocking the process of elastin cross-linking and deposition, and disrupted the normal and orderly binding between elastin and microfiber scaffolds, resulting in loose elastic fiber structure and disordered arrangement (22). Overall, our results confirmed the link between m6A and cell-matrix changes, guiding future research, and elucidating the underlying mechanisms of AD.

Besides, genes with altered methylation at the m6A site are showed enrichment in inflammatory responses, such as the completion of C1r and Tenascin-C (TNC). Among them, TNC is related to cell migration, epithelial-mesenchymal transition/mesenchymal-epithelial transition. The significant expression of TNC in adults is also regarded as a hallmark of injury and inflammation, making TNC suitable for clinical diagnosis. High serum TNC levels at the time of admission of patients with AD are predictors of high mortality, and high serum TNC levels on day 7 of hospitalization might be a low-risk predictor for the expansion of chronic aortic disease (23, 24). In *in vitro*, TNC might play a role in cell migration, inhibition of focal adhesions, promotion of proliferation, and induction or protection of apoptosis. TNC induces innate immunity and up-regulates proinflammatory cytokines and chemokines (25). *In vivo* studies have shown that TNC deficiency can reduce experimental inflammatory diseases such as angiotensin II-induced myocardial fibrosis (26). These findings indicated that TNC reflects inflammation and disease activity, but its exact role in AD progression is still unclear. Based on the results of our study, we speculated that inflammation-related mRNA methylation might affect its expression, leading to changes in the overall translation, regulating inflammation and immune responses, which might be another layer in the mRNA methylation affects during AD formation and development.

In summary, we determined, for the first time, the genetic alterations in m6A regulatory genes in AD, and provided new insights into the pathogenesis of AD by m6A methylation. Further research on m6A target genes in AD might contribute to the clinical application of AD molecular targeted therapy.

## Data Availability Statement

The datasets generated for this study can be found in online repositories. The names of the repository/repositories and accession numbers can be found at: https://www.ncbi.nlm.nih.gov/, GSE147028.

## Ethics Statement

The studies involving human participants were reviewed and approved by the Ethics Committee of Guangdong Peoples' hospital. The patients/participants provided their written informed consent to participate in this study.

## Author Contributions

TS designed and conceived the study. XZ and ZC analyzed the data and drafted the manuscript. RF provided advice and technical assistance. JZ and YL revised the manuscript. All authors contributed to the article and approved the submitted version.

## Conflict of Interest

The authors declare that the research was conducted in the absence of any commercial or financial relationships that could be construed as a potential conflict of interest.
