# Supplementary material for: Transcriptome and N6-Methyladenosine RNA Methylome Analyses in Aortic Dissection and Normal Human Aorta
Source: Front Cardiovasc Med. 2021 May 28;8:627380. doi: 10.3389/fcvm.2021.627380 (PMC8193080; doi:10.3389/fcvm.2021.627380)
Supplement: Supplementary file 1 [file Table_1.docx]

Supplementary Table 1. PCR primers for quantitative real-time PCR.

| Gene name | Abbreviation | Forward primer | Reverse primer |
| --- | --- | --- | --- |
| Methyltransferase Like 3 | METTL3 | TTGTCTCCAACCTTCCGTAGT | CCAGATCAGAGAGGTGGTGTAG |
| Methyltransferase Like 14 | METTL14 | GAACACAGAGCTTAAATCCCCA | TGTCAGCTAAACCTACATCCCTG |
| YT521-B homology F1 | YTHDF1 | ACCTGTCCAGCTATTACCCG | TGGTGAGGTATGGAATCGGAG |
| YT521-B homology F3 | YTHDF3 | GCTATCCACCTAGTTCTCTTGGG | ATGCCAGGCACCTTACTCAAA |
| Fat mass and obesity associated protein | FTO | AACACCAGGCTCTTTACGGTC | TGTCCGTTGTAGGATGAACCC |

PCR primers for quantitative real-time PCR which used in this research.
