# Supplementary figures and images for: Transcriptome and N6-Methyladenosine RNA Methylome Analyses in Aortic Dissection and Normal Human Aorta
Source: Front Cardiovasc Med. 2021 May 28;8:627380. doi: 10.3389/fcvm.2021.627380 (PMC8193080; doi:10.3389/fcvm.2021.627380)

The canonical m6A DRACH motifs of AD and Normal.

**Normal:**


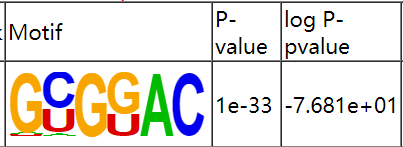


**AD:**


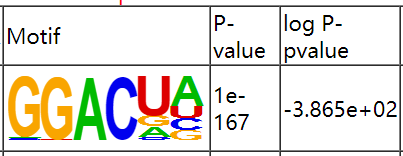

Supplement: Supplementary file 3 [file Table_3.docx]
